# ‘That’s kind of under my work blanket’—redeployment experiences of children’s hospital staff during the covid-19 pandemic: a qualitative interview study

**DOI:** 10.1186/s12913-024-12084-8

**Published:** 2025-01-08

**Authors:** Paula Kelly, Susie Aldiss, Jo Wray, Cecilia Vindrola-Padros, Faith Gibson

**Affiliations:** 1 ORCHID Centre for Outcomes and Experience Research in Child Health, Illness and Disability Great Ormond Street Hospital for Children NHS Foundation Trust, London, UK; 2 School of Health Sciences, University of Surrey, Surrey, UK; 3 Rapid Research Evaluation and Appraisal Lab (RREAL), Department of Targeted Intervention, University College London, London, UK

**Keywords:** Redeployment, Qualitative research, Covid-19, Health professionals, Interviews, Children’s Hospital

## Abstract

**Background:**

During COVID-19 pandemic, a rapid readjustment to continued delivery of healthcare was required. Redeployment is an intentional process to mobilise human resources by reassigning a healthcare worker to a new role or new work location, to achieve sustainable delivery of patient care. We report redeployment experiences of staff from a specialist children’s hospital during first and second waves of the United Kingdom COVID-19 pandemic.

**Methods:**

This study focuses on a specialist children’s hospital where redeployment occurred externally to adult intensive care units and other COVID-19 specific initiatives, and internally as some service activity reduced and others expanded. This was a study of staff experiences using a qualitative rapid appraisal design. Hospital staff participated in an in-depth one-to-one telephone interview. We used a semi-structured interview guide, and recorded and transcribed all interviews. Rapid Research Evaluation and Appraisal Lab sheets were used to share data; team-based analysis was facilitated using a framework approach.

**Results:**

Recruitment and interviews took place from March-November 2021. Twenty-four staff participated: 17 nurses, five doctors and two other healthcare professionals. Interviewees articulated their experiences of redeployment both within the specialist children’s hospital and externally to other health settings (predominantly adult intensive care). Redeployment impacted staff in multiple ways professionally and personally and was reported to be both challenging and rewarding. The reality of working in different settings was felt by everyone, with unfamiliar environments, patient safety, and delivery of a more task-based model of care creating some negative experiences. We identified five main themes: (i) Drivers and Agency; (ii) Journey to Redeployment; (iii) Working Reality; (iv) Personal Impact and Support; (v) Professional Disruption.

**Conclusions:**

Although experience of those redeployed varied, prior background of working in intensive care settings and with adult patients, with opportunities to share practice with colleagues, mitigated challanges for our participants. Positive experiences were associated with perceptions of support received, being welcomed in the new setting, and feeling valued. This study also highlights the act of ‘volunteering’, the nature of ‘voluntariness’ and the difference that may exert on the overall redeployment experience. Future guidance would be encouraged to consider the voluntary nature of redployment.

**Supplementary Information:**

The online version contains supplementary material available at 10.1186/s12913-024-12084-8.

## Background

During the COVID-19 pandemic a rapid readjustment to the continued delivery of healthcare was required. In the United Kingdom (UK) National Health Service (NHS), a range of interventions were implemented to adapt hospital capacity to meet changing demands. These included managing hospital admissions by cancelling elective surgery, and increasing capacity, through for example return of former healthcare staff, use of private hospitals, establishment of field hospitals, and redeployment of healthcare professionals [1]. The unprecedented increase in demand for intensive care services during the pandemic resulted in the fastest and most significant repurposing of services in the history of the NHS [2]. Redeployment, as a government strategy, was an intentional process to optimise and mobilise human resources by reassigning a healthcare worker to a new role or new work location, to achieve sustainable delivery of patient care [3]. Redeployment was used to achieve the sustainable delivery of patient care across the NHS, to facilitate daily work of intensive care units and help to address staffing gaps caused by staff sickness and previous vacancies [4]. The task posed to hospital leaders was enormous, guidance was issued April 2020, with an emphasis placed on safe redeployment, as staff could be working beyond their existing scope of practice, or in contexts that may be unfamiliar [5]. This national strategy was variously established, and depended on the context of particular NHS organisations. Alongside the guidance, from NHS England, professional organisations such as the Royal College of Nursing (RCN) developed a checklist to guide redeployment that prioritised the need for individuals to assess their situation, consider if their employer had met their responsibilities and deciding on what further support might be needed for them to fulfil their role [6]. Emergency powers were implemented to expand the healthcare workforce through regulatory bodies such as the General Medical (GMC) [7] and the Nursing and Midwifery Councils (NMC) [8]. Healthcare professionals had to navigate a complex, regulatory [9] and employment situation during a health emergency, with significant professional and personal implications. Health services attempted to deliver COVID and non-COVID care concurrently, requiring a re-think of staffing models and a re-distribution of existing staff to ensure equitable spread of the workforce to deliver care.

At a more local level in North Central London, in response to the unprecedented increase in the demand for adult intensive care services, partner hospitals across the North Central London collaborated to develop a Central London Sustainability and Transformation Plan (STP). At our own institution, we expanded the referral criteria to a specialist children’s hospital to admissions for general paediatric care, including acute child and adolescent mental health, to enable expansion for adult bed provision in our partner hospitals [10]. We also expanded our Intensive Care (PICU) beds from 44 to 50 and created a dedicated COVID-19 PICU. In response to the national strategy and identified need within North Central London, redeployment occurred externally to adult ICUs and other COVID-19 specific initiatives, and internally as some service activity reduced and others expanded. Staff moved to fill gaps within the hospital and some were released to work out-with their employing organisation. We took part in a series of multisite research studies to gather perceptions of hospital staff working during the pandemic in a range of settings. These studies contributed early findings when considered alongside a rapid policy review and an analysis of media reports [11, 12]. Reported here is the range of redeployment experiences of staff working at a specialist children’s hospital.

## Methods

Data were gathered as part of a programme of ‘COVID-19 Mirror Studies’, led by the Rapid Research Evaluation and Appraisal Lab (RREAL) [13, 14]. Timely reporting was made possible through the work of this Lab, evidenced through reporting on experiences across 20 countries [12]. This team have well-developed skills in adapting research designs and implementing findings at a pace to match the ‘real-world’ of practice [15]. Our contribution to these studies was to undertake a discrete study on the experience of a particular group of staff, staff working in an inner-city specialist children’s hospital. We adopted their approach to methods, data collection and analysis. This involved an iterative process of collection and analysis, where ‘researchers begin with information collected in advance, and then progressively learn from each other and from information provided by semi–structured interviews’ [16].

Staff recruitment was targeted at clinical/service leads to reach out to their staff. We used purposeful sampling across a range of staff, anticipating data saturation would be achieved with 26–30 interviews, based on the experiences of the wider RREAL teams in other UK clinical settings. Participants took part in an in-depth one-to-one telephone interview, these were audio-recorded with participant consent and transcribed verbatim. The interview schedule was adapted from the version used by the RREAL team, with questions specific to the delivery of care to children in hospital (see supplementary file). Data were collected by four researchers (SA, FG, PK, JW), all with significant expertise in undertaking interviews. Informed consent was gained prior to each interview.

### Data extraction and analysis

Under an agreed General Data Protection Regulation-compliant contract, transcripts of digital audio recordings were made by Essential Secretary [17]. These were stored on a password-protected research site-specific shared drive. Anonymised interview transcripts were only accessible to our research team.

Immediately following each interview, researchers completed an RREAL Rapid Assessment Process sheet summarising responses to the interview questions and significant quotations [13]. We used teams-based framework analysis [18, 19], where data from subsequent interviews were added to each researcher’s RREAL sheet. Team reviews of RREAL sheets led to the development of an initial coding framework by two researchers (SA, PK) who further developed and refined this through an in-depth examination of RREAL sheets and review of interview transcripts. We refined the framework as we went along, and went through a number of iterations. This we did through weekly researcher discussions of RAP sheets that ensured ongoing familiarisation and engagement with the emerging data set, enabling all the research team to review the coding and check for accuracy of the interpretation. We looked for differences in redeployment experience and impact within individual participants accounts and across the data set, considering professional background, length and place of redeployment, previous experience of adult and/or ICU care. We largely found shared experiences across the professional groups and thus overall did not feel coding professional groups, such as nurses and medical staff separately was warranted. The resulting themes and sub-themes supported by illustrative quotations, were agreed by the whole research team. The iterative nature of the analysis, and our team discussions allowed us to see patterns and relationships and we realised the significance of redeployment for our participants.

The sample used for this specific analysis consists of 24 interviews carried out between March and November 2021. Interviewees articulated their experiences of redeployment both within the specialist children’s hospital and externally to other healthcare providers. The sample included 17 nurses (NHS bands 6–7), five doctors (all consultant grade) and two other healthcare professionals (Table 1).

**Table 1:** Participant and redeployment characteristics

**Characteristic**	
	**Recruited, n (%)** **(*n* = 24)**
**Area of work to which redeployed during COVID-19**	
Intensive care-(Adult and Children’s)	22 (92)
Other Clinical Services(community responder team, clinical management services)	2 (8)
**Professional group**^**a**^	
Nurses (NHS bands 6–7)	17 (71)
Medical staff (all consultants)	5 (21)
Allied Health Professionals and other staff^b^	2 (8)
**Duration of employment at hospital**^**a**^	Median: 11 years (IQR:5–16 years)
**Gender**^**a**^	
Female	19 (79)
Male	5 (21)
**Ethnicity**^**a**^	
White British	17 (71)
White European	3 (12)
White Other	3 (12)
Black, Asian, Minority Ethnic	1 (4)

^a^We did not sample based on these criteria; data are shown to provide further descriptive information about the sample recruited

^b^Other staff – for example domestic and portering staff, social workers, school teachers and play staff

### Ethical considerations

Health Research Authority (HRA) approved the programme of COVID-19 studies (IRAS: 282,069). Our study was approved through an amendment to HRA and the hospital Research and Development office.

### Patient and public involvement

There was no patient and public involvement for this study.

### Findings

Interview recordings ranged from 25–79 min, with most lasting around one hour. Periods of redeployment spanned the spring 2020 and winter 2021 waves of COVID-19 in the UK. Some participants were redeployed during both waves and others for a period during the first or second wave only. Length of redeployment ranged from a few days to four and a half months. Participants who were redeployed within the hospital mainly moved to work within paediatric intensive care settings, where care included children with COVID-19 and later paediatric inflammatory multisystem syndrome temporally associated with SARS-CoV-2 (PIMS-TS). Other internal redeployment roles included clinical management services. Participants who were redeployed externally moved to either Intensive Care Units (ICUs) in North Central London to care for adults with COVID-19, community responder teams or the Nightingale Field hospital [20]. Many of the nurses and all the doctors who went to work in adult ICU had ICU experience, although mainly with children. Some participants had never looked after adult patients before or worked in an ICU environment. The majority of nurses were registered as Children’s Nurses (NMC register). Eight participants reported taking on additional roles, as a contribution to the increased need for healthcare staff, such as joining vaccination teams or undertaking bank shifts on adult or children’s ICU. They tended to be staff who were unable to be released from their current role to be redeployed elsewhere and utilised annual leave and days off to help in some way.

**Fig. 1:**
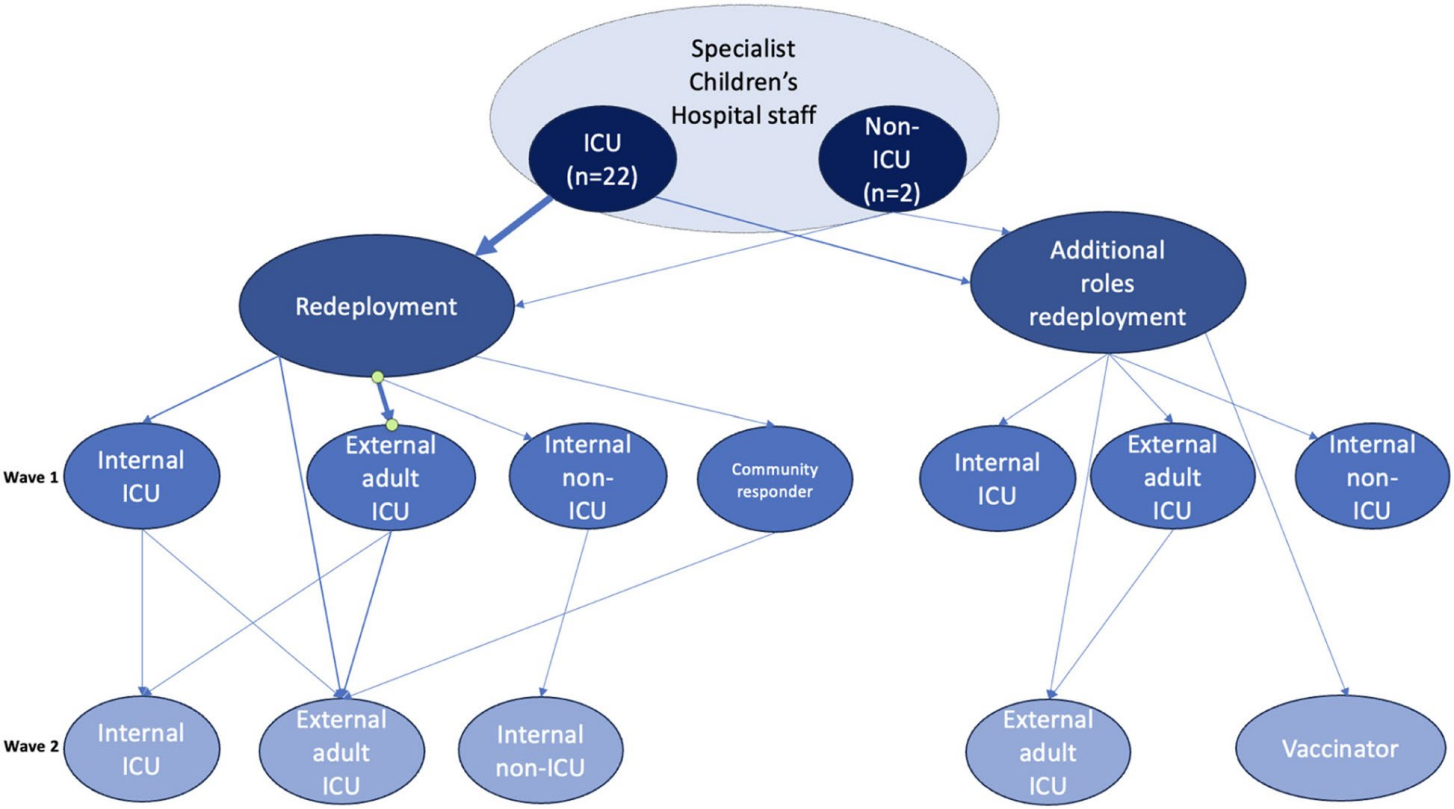
Complexities of redeployment. Footnote: Line thickness denotes frequency – i.e. number of staff on each path of redeployment; in wave 1 two members of staff were redeployed to a Nightingale field hospital before being redeployed to an adult intensive care unit

Figure 1, illustrates the type, frequency and location of redeployment.

### Themes

**Fig. 2:**
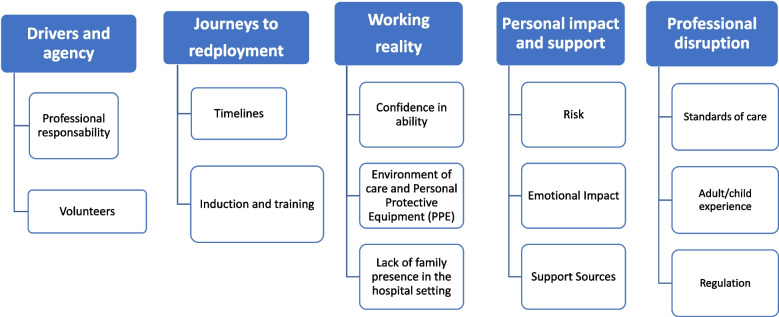
Themes and sub themes

We identified five themes from analysis of staff redeployment experiences: (i) Drivers and Agency; (ii) Journey to Redeployment; (iii) Working Reality; (iv) Personal Impact and Support; (v) Professional Disruption (see Fig. 2). We present these themes with illustrative quotes where relevant.

#### Drivers and agency

Participants discussed what drove them to volunteer for redeployment, with many describing a strong desire and a feeling from within to help their colleagues and patients in this health emergency.

##### Professional responsibility

The drivers for being redeployed included: a recognition of the seriousness of the situation, wanting to ease the impact upon colleagues in adult ICUs and, as health professionals, feeling a moral and professional obligation to use their skills by going to help,

*“I just remember thinking about the nurses that were working there and just thinking, if I were one of those nurses I would be exhausted. And I don’t know how much I can help but if I can make one nurse, if I can make one shift for one nurse slightly better, then it’s worth, kind of that’ll be worth it.”* (G04, Nurse)

Many participants stated that they wanted to do something positive, to learn, and would have felt helpless and guilty if they had not acted and been redeployed,

*“I think had we stayed at……..(specialist children’s hospital) looking after children I would have been worse off, because of the frustration it could have brought about, and helplessness if you like. So, I’m quite proud of the fact I went and worked with adults and quite grateful for the experience that I faced at the time. I certainly learnt bits and it broadened my horizons and I made friends and relationships with a new bunch of people*.” (G21, Doctor)

There was both a recognition and a willingness to do so, they did not need or in some cases wait to be asked, staff were in control of this decision and took action accordingly.

##### Volunteers

Most participants expressed the voluntary nature of their redeployment decisions, the hospital asked for volunteers, external redeployment was not mandated by our organisation, staff were able to exercise agency,

*“Whenever I was going I was volunteering to go it never got forced upon me.” (G05 Nurse)*

The agency that staff felt was illustrated by their reports of mangers supporting them in their decisions, including having the option of not continuing with a planned redeployment if the experience was traumatic,

*“My manager was quite supportive and said that we could go over for however long we wanted. She was very supportive in saying to us that if we were unhappy after the first shift we just needed to say and come back.” (G19 Nurse)*

Some participants also described opportunities to influence where they were redeployed (including internal redeployment) because of concerns for a vulnerable family member (e.g. due to pregnancy, age or underlying health condition),

*“My first shift in (Paeditric) ICU, I felt good because I was helping in the situation, because of all the pandemic and I was feeling that I wasn’t doing too much considering what the other nurses do and I felt proud about them, but I said I’m a nurse in this pandemic and I’m not doing anything ….. Because I was feeling guilty before that and a lot of my colleagues went to help in another ICU and I didn’t go because I have child with a ……(health problem) and I didn’t want to risk it.” (GO6 Nurse)*

In addition to helping ease the burden on overwhelmed colleagues, participants expressed a strong pull to help patients, for example one participant described thinking about how if they had a family member on ICU, they would want them to have good care, not care that was compromised due to short staffing. Participants identified multiple factors including personal, skill sets, professional and moral imperatives to do what they felt was the right thing and volunteer for redeployment, both within their own hospital and externally.

#### Journeys to redeployment

There were variations in the journeys participants experienced for redeployment into adult ICU and to other services and teams within their own specialist children’s hospital.

##### Timelines

There were differences in the timelines and processes to redeployment for staff, including further changes across the first and second waves. For some who responded to the hospital executive or their line manager’s request for people to volunteer to be redeployed, there was a time lag waiting to find out if they would be selected and where they would go. They expressed frustration at the uneven flow of information from the hospital team managing this,

*“There was an email that had been sent round to say, ‘Would you volunteer, …. you don’t have to but if that would be something you would be interested to do leave an expression of interest as an email.’ And I did that and then, I don’t know that actuality, but I feel like there was at least a couple of weeks and I heard nothing ….. and then it was last minute, and it was, ‘All right you can go and you’re going to go next week’, and then just like that I was having my induction” (G20 Nurse first wave)*

During the second wave communication lines were better established, leading to more rapid decision making,

*“We got the briefing I think on New Year’s Eve that said if anybody is happy to go and help in adults then it would be gratefully received so me and a couple of my colleagues said, ‘Hands up we have no adult experience but are happy to go but please do not send us to intensive care,’ and then an hour later I got a call from the matron at (adult hospital) ICU saying, ‘Please can you come tomorrow.’”(G16 Nurse second wave)*

Some of the medical staff were proactive in contacting adult ICU colleagues and offering their services,

*“I have some collaborations with the guys who work there and I basically said what do you need? Because at the time the PICU (Paediatric Intensive Care Unit) was very quiet.” (G23 Doctor)*

##### Induction and training

Training and education for the redeployment setting varied. Some hospitals had a short formal induction, that in the second wave, became more organised, other participants experienced a period of being supernumerary or undertaking roles with less responsibility for decision making.

Induction/training was described in the first wave as ‘chaotic’, with the predominant experience tending to be very brief due to the ratio of staff to patients. Shared feelings of being left to get on with the role and quickly learning to undertake many new tasks,

*“It was really, really busy and the nurses were really like under a lot of stress and a lot of pressure and so I didn’t really, I think I probably could have asked for more help but I didn’t want to make them, I didn’t want to put pressure on them to like teach me things. So, I just tried to like pick things up myself and I would ask the other support workers just little things, or they would help me kind of find my way around and work out where certain things were …….. but it was pretty tough.”* *(G07, Other staff)*

*“I was shown around, I was shown where the staffroom and the toilets were and stuff, that was about it, yes, I didn’t really get any sort of any induction or any information from (specialist children’s hospital). I just was given the name of a person at (hospital) to contact and she just gave me like the shift times and the location.” (G14 Nurse).*

This contrasted with other participants’ experiences of a more comprehensive induction-despite high patient workloads in the second wave redeployment,

*“I thought we would be thrown in, ‘you’ve just got to crack on, we’re really busy’, but actually we had a whole morning. They went through the paperwork, they went through what their monitors look like, their observation monitors, they fit tested us all, they showed us round the unit and, yes, I felt the orientation was really good actually and it wasn’t rushed.” (G16 Nurse)*

Although information flow was uneven, particularly during the first wave, the consensus of many redeployed staff was that in this unprecedented health emergency bespoke preparation was challenging to provide in a largely unknown and rapidly changing situation for all healthcare providers. In our cohort, no specific information about adult care and treatment was described as part of any preparation initiatives.

#### Working reality

As staff reflected on their preparation experiences, it was clear they were faced with a range of challenges through working in new places, with different patient cohorts and managing complex infection control processes. The working reality of redeployment, particularly to adult intensive care, included higher patient ratios to staff, repurposed spaces being used to treat intensive care patients, the physical and communication challenges of Personal Protective Equipment (PPE) and prioritisation of essential care.

##### Confidence in ability

Participants redeployed into adult ICU reported a range of confidence in delivering care. Some felt supported—that the ICU staff were grateful for their help, they could ask for help if needed, and say if they did not feel confident to do something. Others felt overwhelmed and constantly out of their depth, they were left to care for very sick patients without the support they needed,

*“I felt like I was just treading water for the entire day and I hadn’t really got my head above water at all, and that was every single shift, and that was quite tough in that sense.”* (G19, Nurse)

*“At the beginning we were just handed a bunch of patients who were obviously very different from our normal patients to look after. So, it was all cobbled together, but everyone else was making it up as they went along as well.”(G22 Doctor)*

In addition to COVID-19 being a new disease and uncertainty about how best to treat it, there were many aspects of redeployment which were uncertain and added extra stress. One participant described every day as being like their first day as they moved around different units and so did not know the staff or where any equipment was kept. There were high patient to staff ratios and the physical burden of care was challenging. This meant they were unable to offer the level of care to patients that they would usually, instead essential care had to be prioritised,

*“…so I’d have a bay of four patients and I can’t tidy up and I can’t clean their teeth because all four of them need drugs. All four of them need suction…I have to prioritise and unfortunately, you know, changing a bed sheet doesn’t come above steady medication but it is irritating. It feels, when you go home, it feels incomplete that you haven’t quite done your job that day.”* (G09, Nurse)

##### Environment of care and personal protective equipment

The environment made the work more challenging as spaces which were not usually ICU areas were repurposed. This meant spaces were cramped and disorganised, there was often not enough space between beds which impacted upon patient’s privacy, for example when they were being washed,

*“An area where I was first put was a theatre…. And I just remember walking in and thinking this is not where - this space is not meant for this kind of care. It was cramped. It was small. There were too many patients in such a small area. …..theatres are not made for that kind of, those sort of care, we were having to squeeze around spaces, equipment was available but not in the areas you needed it because those spaces had been turned into extra beds in such a short space of time.” (G04, Nurse).*

Working in PPE made the work more exhausting as it was hot and uncomfortable. It also impacted on communication between staff and with patients. It was often impossible to identify staff and their role. Some units had developed a colour code or letter system to make it easier to identify staff roles. Participants found this to be reassuring as they could identify who they could ask for help and their own role was made clear to others, so people were less likely to ask them to do tasks they could not do,

*“….we were categorised into A, B & C and they were very fair, they categorised us as C - category A was intensive care experience, category B was Nurse and category C was HCA (Healthcare Assistant) so because we were children’s nurses, they put us into that category. They gave you very clear job roles that were expected, which gave you a bit of a safety net that you were not going to be in at the deep end!”* (G16, Nurse)

##### Lack of family presence in the hospital setting

Many participants commented on how unusual it was to care for patients who were ventilated and did not have any family present at the bedside, this made it harder to get to know them. One doctor described how regular phone calls with family were important to help them build connections with patients,

*“…people there, it was kind of very difficult not to treat them formulaically so I was very keen to have phone calls with relatives and I did that, updated them at the end of the day and that was very, very helpful. Getting to know them as people rather than just the bloke in bed four who’s got COVID like everyone else has got at the moment and that was potentially - I found that a little tricky and I had to work at just making sure that you treat people as individuals.”* (G17, Doctor)

Staff redeployed within the children’s hospital also referred to restricted family visiting (only one parent) echoing both the difference from usual practices of family-centered care and the additional workload for staff who are normally working alongside parents in the delivery of care and support,

*“It was very challenging because some of the children and little ones never had any of their family members with them like at certain times. So it was quite upsetting for the kids because obviously nurses and nursing staff and doctors were trying to do things to the patients. And they don’t have their family there so they become upset.” (G42, Other staff).*

Providing end of life care with no or restricted family members present in the care setting was highlighted by several participants as very challenging. This included participants working in adult ICU as well as those in the paediatric setting,

*“I remember an older man, similar age to my dad, and he was at end-of-life care, unfortunately, and his family were only allowed to FaceTime him, to say their goodbyes. That was really, really tough. I found that really, really─ I think we all found that─ the whole nursing team on that day found that really upsetting. And that has stayed with me the whole time really.”* (G19, Nurse)

#### Personal impact and support

Participants discussed their perceptions of the risks to themselves and their families, how redeployment impacted on them emotionally, how they coped and the various ways they sought support.

##### Risk

At the start of the first wave, some participants did not necessarily think they were at high risk, at this time, it was generally thought that older people and people with underlying health conditions were at most risk of serious illness. This changed during the second wave with younger and previously healthy patients admitted to adult ICU, patients more like the redeployed staff, which made some participants more anxious about the impact of COVID-19 infection on their own well-being,

*“I mean over Christmas and New Year (2020/21) it just seemed to go crazy and there was all the talk of a new variant. So, I think, you know, and younger people getting sicker and I think, you know, we couldn’t, well I certainly couldn’t* *,* *that kind of idea of it being older people or people with underlying health conditions was long gone.” (G02, Nurse)*

Some participants also reported fears for personal safety, in the deserted city centres when traveling to and from work,

*“We didn’t feel safe around the hospital at that time –other colleagues reported who were getting the tube felt unsafe –it was very eerie after finishing work at eight or nine in the evening. There was no-one around …reports of mugging of NHS staff so deserted.” (G03 Nurse)*

Throughout all participants reported being concerned about the risk they could pose to their own families and described a range of measures they took to safeguard them. Their concerns included being exposed to COVID-19 at work or when using public transport travelling to/from work. Some staff stayed in a hotel during their working week, to protect their family and their patients, several reported a strict routine of washing/changing clothes as soon as they got home.

Family members also worried about them working with COVID-19 patients and the risks,

*“My husband was very unimpressed. I think his comment was, ‘I don’t understand why you would volunteer to do this. You are safe at (specialist children’s hospital).’” (G18, Nurse)*

##### Emotional impact

The daily emotional impact of working in clinical settings during the pandemic was high, particularly so in adult ICU,

*“It was more the family members that would phone up and the emotional side of things, you know. Witnessing a lot of deaths, witnessing a lot of family separation, and isolation, and all the ─ and also there was quite a lot of what they called “possible ICU psychosis”. That was quite tough to watch as an adult because, you know, I’ve seen children go through it but I can cope with them, but with adults it felt, you know, that could be my dad, that could be my mum, and it just felt all very relatable and very close to me as an individual.” (G19, Nurse)*

For many, the emotional impact became apparent once they had finished working on ICU. Some experienced flashbacks and had trouble sleeping. At the time, they were so busy and exhausted they focused on the present and getting through each day. It was only later, looking back and thinking about the people they had cared for, the people who died and the sadness of the situation that they released how distressing it was,

*“At the time it was fine, you know I look after very sick patients in very stressful situations now in my day-to-day job but I think with hindsight I recognise how difficult it was with periods of trauma, because you get in and get going and just make the best of it and then it’s only when you step off the treadmill afterwards that you realise quite how abnormal it was.” (G22, Doctor)*

Others described strategies they developed to cope, such as exercise and compartmentalising their work and home life,

*“If I sit and really think about it, and when it was at its peak and all them people dying … because …..that’s kind of under my work blanket, I work in intensive care, people die and actually I take some pride in even when they’re really sick I’m doing the best I can for this complete stranger. So I think I can kind of rationalise it and also say, this isn’t my, I mean this is a sad situation that person is going through this but it’s not my sad situation but theirs.” (G20, Nurse)*

Many participants also described a positive effect on them, they were glad that they were not stuck at home, were able to continue to work and pleased that they were able to help. Many said that they would volunteer for redeployment should the need arise in future.

##### Support sources

In articulating their support needs and the choices they made on seeking support, participants described a complex range of needs and preferences. Support was offered for all staff during the pandemic through a wellbeing hub. This included online group support sessions as well as a group session for staff who had been redeployed on their return to work. Participants were aware of the wellbeing hub, but most did not access it, and some did not feel that group sessions were the right support format for them.

More informal support was usually preferred, this included managers and colleagues ‘checking in’ with them via phone/email/text whilst they were redeployed and in person on return. Being redeployed with other staff members they already knew well was particularly beneficial. ‘Whats App’ groups were also set up between staff who were redeployed to the same setting which provided peer support,

*“We set up our own What’s App group ….. we still managed to talk through stuff and sometimes we would call each other, to discuss it and rarely emergency things it was more observations about how things functioned and what can be done better or differences from what we normally do in our sphere, recognising that and acknowledging that.” (G21, Doctor)*

When the staff in the redeployment setting were supportive, welcoming, and made an effort to get to get to know them, this made the participants feel valued, they felt part of the team and this increased their job satisfaction,

*“Most of the time the staff that we were working with, the vast majority were so unbelievably grateful. They were so lovely and again, there was an amazing sense of camaraderie and the proning team when they came round were just kind of really excellent, like a breath of fresh air and I’d be chatting to them and they’d ask me where I’d come from…”* (Nurse, G02)

For participants who struggled with their psychological wellbeing during/following redeployment, they were able to access further formal individual support, although some felt that this should have been offered sooner.

Practical support offered was much appreciated, such as free meals when on shift, free bicycles to travel to work and free accommodation near the hospital.

#### Professional disruption

Participants described disruption to their usual practice, as some were working in different settings which they were not trained for and in situations that challenged the level of care they wanted to provide. Relaxation of the regulations from professional bodies was also discussed.

##### Standards of care

Participants described how the situation in adult ICU challenged their professional norms and roles,

*“We’d kind of prepared for war in a way… that sounds like a dramatic way of saying it, but I didn’t really know what I was walking into…. You would never be able to just walk into a hospital and basically just go and look after a patient. But that was what happened…” (G15, Nurse)*

Nurses particularly described how they were unable to give the usual level of care to a patient, which they found distressing,

*“You know, basic nursing care I wasn’t able to achieve and that really ─ I think that upset me the most, on most days. Other days it was because of the intensity of it all and the sickness, but every single day it was because I wasn’t achieving basic nursing care, which I just─ isn’t, you know, it’s not part of us as nurses. You know, we all ─ We all have it drilled into us and we all know the best thing to do for patients is to deliver basic nursing care and we couldn’t do that as a team, at all really. That’s why I struggled the most, to be honest.”*** (G19, Nurse)**

A doctor described how their focus had to change from improving health to keeping people alive,

*“I mean most of the time what you were doing was stopping them dying rather than making them better, if you can follow the distinction.” (G23, Doctor)*

For participants who were redeployed within the hospital to work with children in ICU, they also discussed that were unable to care for patients in the way they were used to, due to PPE which impacted on communication, the absence of parents and the level of distress of some children.

##### Adult/child experience

Participants who had worked with adult patients tended to feel more confident about their ability to care for these patients. Some nurses had previous experience and training in the care of adults including in an intensive care setting, which had to some extent motivated them towards redeployment. For medical staff, the majority described their adult experience as historical and some expressed initial concerns about different co-morbidities to their paediatric intensive care patients,

*“So, we were quite worried about a lot of things, heart disease or COPD (chronic obstructive pulmonary disease), adult medicine. But actually, I was quite relieved to find out that most of those (patients who) had come across our radar was pretty much (the issues) what we dealing with was the same as on a paediatric ICU. So, the problems are the same and the medicine was actually pretty similar. So, we kind of fell into a routine very quickly.” (G21, Doctor)*

Many of the nurse participants were trained to look after children and not all had previously worked on ICU, looking after adults in this environment was new and some of them felt anxious,

*“I guess I felt quite scared and I was also worried because it wasn’t my scope of practice. You know, I’m kind of registered as a children’s nurse and I was really worried that I didn’t have the skills to be able to do the right thing looking after adults.” (G01, Nurse)*

Some nurses described how different it was looking after adults to children. Some aspects were easier, such as the drug doses being consistent,

*“I was very aware that the medicines are very different and they give them in a very different way, and after a while you realise that actually it’s easier than children’s because they have the whole vial or the whole tablet….so it took a while to get your head around it. But I did feel like I had that space to wait, assess who I was working with and if I would have support from them in that role.” (G16, Nurse)*

However they were also confronted by care issues they had not faced before, which were more difficult and made them anxious about getting things wrong, such as talking with adult children of patients and shaving a patient.

##### Regulation

Medical and nursing staff were aware of the “relaxation” in scope of practice made by their professional bodies, however this did not necessarily resolve their anxiety to make sure they were doing the very best for patients,

*“I knew that the RCN and the NMC had sort of said that they would allow you to go and work in other places and support you from that instance. But I definitely did feel out of my depth sometimes in terms of not really knowing if the policies were the same, and not really having anyone to ask or anywhere to look to see if that was the same.” (G15, Nurse)*

Resolution of these dilemmas came both from the support they received from colleagues and drawing on their clinical decision-making skills and experience,

*“It’s working outside your scope of practice which as you’ll know you can’t do that, the GMC, the rules tell us we have to work within our scope of practice, then you get a pandemic and it’s like, oh no, you’ll be fine, carry on, that was a bit weird. Actually we had great support from the guys, the adult intensivists we were working with, so never felt particularly vulnerable that way ………” (G17, Doctor)*

For some staff these disruptions were a personal and professional learning opportunity, they helped them to reconnect with the fundamentals of their identity as a healthcare professional.

## Discussion

Redeployment was a national strategy to mobilise staff, to have the right staff in the right place and enough of them to manage the ever-increasing number of very sick adults requiring intensive care. This, within a climate of operating in environments of scarcity, was in place long before COVID-19 [21]. How this was operationalised varied, reflective of the complexity of the NHS, chronic staff shortages and hospital and community Trusts making the best decisions they could, responding to need based on limited evidence of what was unfolding. The experience of those redeployed was bound therefore to be varied, and there is much to learn from these various experiences in published work, some of which has been mirrored here, and will be considered briefly prior to our reflections on the nature of voluntariness, when faced with a national call to redeploy staff. This was experienced very differently by staff in our children’s hospital and contrasted to elsewhere, where for example preliminary guidance was asking for a process to manage nurses who refuse to be deployed [22].

Staff who experienced redeployment, external to and within a specialist children’s hospital articulated the context of their experiences during periods of the COVID-19 pandemic when patient hospital admissions were at their greatest numbers and highest levels of morbidity and mortality. At the outset, the narrative was around building competence and confidence, putting in place supervision, training and ongoing support. This was in order to counteract the well-known fact that redeployment of staff to another area creates anxiety, with staff feeling no longer in control. This anxiety was increased during COVID-19 by fears of infection, that this would be transferred to other staff, patients or family members. The working reality of providing clinical care in these circumstances was challenging. Impact on staff was felt both personally and professionally. This is reflected in the concerns others have raised about the reduction or absence of family visiting in adult and children’s hospital settings [23, 24]. Our participants, similar to other reports, reflected on preparation for redeployment, their expectations, apprehensions and concerns [25]. Familiarity, for those skilled in intensive care, lessened the burden for some, but those not used to caring for adults faced different, sometimes more practical challenges. The reality of working in different settings was felt by everyone, with unfamiliar environments, patient safety and the delivery of a more task-based model of care creating more negative experiences, as has been described elsewhere [26]. Consistent with existing literature, positive experiences were also described by our participants, where support was tangible, informal rather than formal, and staff were welcomed and felt valued [27]. Interestingly although the majority of our participants voiced no regrets taking part in redeployment, there remained concerns related to the organisation of redeployment, including the impact of services at the children’s hospital [28] and the longer-term implications of staff on their own work and returning to roles within their teams. Like others, the implications of the redeployment process was not consistent, the absence of policies for healthcare during a crisis and variation in practices was felt most keenly by those redeployed [26].

The nature of redeployment in a crisis have been echoed in the literature reflecting mainly adult healthcare staff experience [25]. Health visitors and other community-based staff, particularly in mental healthcare articulated the abandonment of vulnerable caseloads, without sufficient time to make any provision or safety netting. In the main what has been described was a mandated approach from employers and managers. Abrams et al. [29] reported nursing staff as feeling they were in a “no choice” situation with those who expressed concerns about how their organisations managed redeployment, being characterised as “contentious” and political”. The overall result was feeling under threat and knowing that speaking up was futile. This contrasted with the majority of our participants who expressed having volunteered for external redeployment to adult services particularly intensive care. These participants felt a moral duty to help in such an acute healthcare crisis, echoing the results from a UK survey of 240 nurses [30] where the majority felt it was their duty and were prepared to work where they were asked to and were needed. However, they also expressed a lack of choice associated with details and the process of redeployment. It would seem that most of the participants in our study experienced more of a sense of volunteering, in some cases in response to a call to volunteer, with some ability to negotiate location and length of redeployment. This qualitative difference in the nature of redeployment is reinforced by examples of “informal” redeployment including staff returning to undertake “bank” shifts in adult ICU’s once they had recommenced their own roles back within the children’s hospital. The recognition of need alongside personal and professional drivers to help is already documented across the literature [11, 31–33]. What varies is how redeployment strategies were put into place and levels of autonomy that staff experienced including a lack of equity amongst some staff groups.

Different to many other studies, our study shines a particular light on the act of ‘volunteering’, the nature of ‘voluntariness’ and the difference that may exert on the overall experience of redeployment. Some participants in our study had volunteered without being asked, others were asked, and some continued to volunteer some of their time having returned to their own work. There were also some participants where ‘the ask’, particularly internal redeployment, fell to more than one person in a team to undertake redeployment, and some staff felt more ‘obliged’ than others to respond to ‘the ask’. Our organisation chose to ask healthcare professionals if they were willing and able to volunteer to be redeployed. Choice was transparent in all of the internal communication, as was decision-making, in terms of the location and length of the redployment. This approach was qualitatively different to other organisations where staff were not given choice about redeployment, the impact of which is known to be highly relevant to experience and features in more recent guidance on redeployment [34]. Professional identity, a sense of duty, professionalism and agency to take action featured in the accounts of those redeployed in our study. Others have described how this creates a morale dichotomy between professional commitment and personal responsibility [35]. Evidence is overwhelming for the need for guidance to support HCPS in their decision making.

Voluntariness can be understood as a choice that is made in accord with a person’s free will, as opposed to being made under coercive influences or duress. Voluntariness also requires that sufficient information is provided to the potential user to make informed decisions. Similar to the donation of blood, or organs for transplant [36, 37] the reasons why some of our participants volunteered before being asked, and others volunteered in response to ‘the ask’, maybe more complex than simple altruism [38]. In-depth motives, ingrained in the sub-conscious, the essence of being human, having a social conscious and values at the core of being a healthcare professional, wanting to help their colleagues, responding to the need of patients in hospital. This might have created a different driving force to step into a new environment, with staff having volunteered in the absence of understanding risk in the immediate and longer term. As Connolly et al. [32] have demonstrated in their longitudinal study the experience of redeployment changed over the course of the pandemic. Understanding more fully the nature and response to redeployment when used as a strategy to mobilise human resources in a pandemic requires us to understand the human factors that shape response.

### Limitations

Data for this study were collected from a single inner-city specialist children’s hospital with a self-selected sample of staff. The experiences of staff from other specialist children’s hospitals, and children’s units which are part of mainly adult hospitals may be different and limit the transferability of our findings. In future pandemic research we suggest the importance of research that seeks the experiences of health care staff working in settings outside of those seeing the largest numbers of seriously ill patients, for example children’s services, mental health services and community health care. The majority of staff interviewed who were redeployed externally expressed the voluntary nature of this, with some choice about location and length of redeployment, and the knowledge they could stop if they wished. There may be other staff who did not feel they had these choices, where perhaps they felt pressured into redeployment, or it was mandated.. These experiences could be more difficult to speak about and so they may not have come forward for interview, thus their views are not represented here.

We worked with the Rapid Research Evaluation and Appraisal Lab (RREAL), using their methods. Slow recruitment to our study however, meant that these ‘rapid’ methods were unable to inform wave 1 and 2 of the pandemic. Should there be a 3rd wave, there is knowledge here that will influence practice.

## Conclusion

Redeployment impacted staff in multiple ways professionally and personally in this study of a single specialist children’s hospital. Managing redeployment more effectively in the future should consider all that has been documented about the experiences of healthcare staff to ensure staff are empowered in their decision making. Planning and responding to future needs for health staff redeployment requires attention to skills and capacity and staff wellbeing, in order to safely harness the drivers that prompt staff to volunteer in a health crisis. Guidelines are now available, informed by other COVID-19 studies, able to inform good practice for redployment outside of a pandemic [34]. Future redeployment programmes would be wise to consider the nature of ‘the ask’, and use these guidelines, as well as consider the implications on teams and settings from which professionals are deployed.

## Supplementary Information

Supplementary Material 1.

## Data Availability

No datasets were generated or analysed during the current study.

## Abbreviations

COPD: Chronic obstructive pulmonary disease
HCA: Healthcare assistant
HRA: Health Research Authority
ICU: Intensive care unit
NMC: Nursing and Midwifery Councils
PIMS-TS: Paediatric inflammatory multisystem syndrome temporally associated with SARS-CoV-2
PPE: Personal protective equipment
RCN: Royal College of Nursing
RREAL: Rapid Research Evaluation and Appraisal Lab
UK: United Kingdom
