# Supplementary material for: ‘That’s kind of under my work blanket’—redeployment experiences of children’s hospital staff during the covid-19 pandemic: a qualitative interview study
Source: BMC Health Serv Res. 2025 Jan 8;25:46. doi: 10.1186/s12913-024-12084-8 (PMC11716227; doi:10.1186/s12913-024-12084-8)
Supplement: Supplementary file 1 — Supplementary Material 1. [file 12913_2024_12084_MOESM1_ESM.docx]

**Supplemental file**

**INTERVIEW GUIDE: HEALTHCARE WORKERS (HCWs)**

Date:

**Respondent Information**

| Gender | Age | Ethnicity | How long in your professional role (years) | Highest Education level | Role/position | Follow-up? |
| --- | --- | --- | --- | --- | --- | --- |
|  |  |  |  |  |  |  |

*“The interview takes about 20-25 minutes on average but it can go on longer depending on how much you want to say”*

**First, I want to ask you about your work and the services you provide. (This section not COVID-19 specific).**

1. **Background: Can you tell me about your role? (Daily tasks, where in the hospital, responsibilities)**

- *Can you tell me a bit about your role?*
- *Please briefly describe your normal daily tasks/responsibilities.*
- *Do you have a carer role outside of the hospital?*

**Now I want to ask you about health services during COVID-19 outbreak.**

1. **Have you been in contact with patients who had suspected and/or confirmed COVID-19?**

*Probes:*

- - *In what capacity?*
  - *How have you found working around these patients?*
  - *PPE physical effects? (E.g. dehydration, muscle ache, irritation)*
  - *What psychological/emotional impact did this have on you?*

1. **How has the COVID-19 outbreak affected health services in your department?**

*Probes:*

- *How has this affected your normal daily tasks/responsibilities? Change of role?*
- *Impact of COVID-19 on the delivery of services to non-COVID-19+ patients (i.e. cancellation of elective surgeries)*
- *Can you tell me about the impact of visiting for family members (parents, siblings, extended family)*
- *Are there any other of the routine patient services for children and families that have impacted (play services, hospital school, activities available in the hospital, access to child orientated spaces)*
- *What tasks are you able to do more or less effectively?*
- *How do you manage the isolation of suspected cases and confirmed cases?*
- *Has there been appropriate transfer of patients within and out of hospital?*
- *Has there been an impact on staff’s ability to make diagnoses and act on them?*
- *Supply of drugs, equipment and PPE?*
- *Redeployment of staff from or within your health facility*
- *Motivation and capacity of staff to work? (probe: exhaustion, fear, etc.)*
- *Psychosocial and moral support – did they receive any? Enough?*

**5. What were the preparedness strategies implemented locally (department, hospital or Trust)?**

*- Did you feel these strategies were enough?*

*-What do you feel was particularly successful?*

*- Should the Trust have prepared differently?*

*-Did you receive any training? (including but not limited to PPE training such as mental health and well-being training)*

**6. Do you currently have any concerns or fears in relation to ...**

- Work
- Your personal life (personal health, family, responsibilities)
- The national effort

1. **Over the past months, have you experienced any problems with:**

- Hours and quality of sleep
- Eating (underrating, overeating)
- Concentration (trouble concentrating when doing simple tasks)
- Self-efficacy (believe in ability to succeed)
- Worry/anxiety (frequency in last week)
- Drinking or drug use (more or less frequent than usual)

1. **Mental health support (to address risk of moral injury, trauma and developing severe mental health problems)**

- Are you aware of any support available for staff wellbeing and mental health?
- Have you had the opportunity to talk about your mental health with your supervisor/team leader?
- Have you had worrying/traumatic experiences in the last week? Did you receive support after? If so, what type of support?
- Interactions between peers: Do you have time to socialise with your team? what has changed with COVID-19?

**9. Are you using local data to inform response efforts?**

-What type of data?

-Who normally collects the data?

-How are data shared?

-How are data used to make changes in practice?

**10. How have health services been strengthened, or how could they be strengthened during the outbreak?**

*Probes:*

- ***Support to HCWs from the health system and partners?***
- ***Capacity for rapid response***
- *Policies? e.g. Guidance and emergency protocols?*
- *What would help HCWs to maintain normal services as well as COVID related services?*
- *If GP: Health promotion and community engagement. How?*
- *If GP: Linkage to other support organisations, e.g. charities, schools?*

**11. Is there anything you feel should be changed to make health services more effective in future emergencies?**

*Probes:*

- ***Support to HCWs? From whom and How?***
- *Coordination and official guidance of COVID-19 response.*
- ***Early detection and reporting.***
- *On-going health promotion and community education. E.g. potential sources of infection, safe practice?*
- *Mobilisation? E.g. identifying and coordinating trusted community volunteers and support?*
- *Disease outbreak control activities?*
- ***Testing (public and staff)***

**12. Is there anything else you would like to mention that you feel is important?**

**Thank you for your time and for sharing your opinions and experiences with us.**
